# Management of fever and acute painful crises in children with sickle cell disease in emergency departments: a tertiary hospital experience

**DOI:** 10.3389/fped.2023.1195040

**Published:** 2023-06-12

**Authors:** Tameem Almahmoud, Tasneem Alnashwan, Lara Al Kuhaimi, Mohammed F. Essa, Nouf Al Balawi, Khaled Al Jamaan, Nesrin Al-Harthy

**Affiliations:** ^1^ Department of Pediatric Hematology/Oncology, King Abdullah Specialist Children’s Hospital, Ministry of National Guard Health Affairs, Riyadh, Saudi Arabia; ^2^ Department of Pediatrics, King Abdullah Specialist Children’s Hospital, Ministry of National Guard Health Affairs, Riyadh, Saudi Arabia; ^3^ King Abdullah International Medical Research Center, National Guard Health Affairs, Riyadh, Saudi Arabia; ^4^ College of Medicine, King Saud bin Abdulaziz University for Health Sciences, Riyadh, Saudi Arabia; ^5^ Department of Pediatric Emergency, King Abdullah Specialist Children’s Hospital, Ministry of National Guard Health Affairs, College of Applied Medical Sciences, King Saud bin Abdulaziz University for Health Sciences, Riyadh, Saudi Arabia; ^6^ College of Applied Medical Sciences, King Saud bin Abdulaziz University for Health Sciences, Riyadh, Saudi Arabia

**Keywords:** sickle cell disease, vasoocclusive crises, fever, emergency management (EM), painful crisis

## Abstract

Sickle Cell Disease (SCD) is highly prevalent in Saudi Arabia with variable demographics and access to health care facilities including emergency departments. Literature reviews for locally published articles are deficient in the in-depth evaluation of current emergency practices in managing patients with SCD. The study aims to assess the current emergency practice in managing SCD patients in tertiary hospitals. We reviewed data of 212 visits by patients with SCD over three years and assessed the current emergency department practices in managing common SCD crises, such as vaso-occlusive (VOC) and febrile episodes. Our findings revealed that 47.2%, 37.7%, and 15% of the patients presented with pain, fever, or both, respectively. The patients were triaged level III according to the Canadian triage and acuity scale system in 89% of the visits. The Median time for patients to see healthcare providers was 22 min. In the first 2 h, 86% of the patients received at least one fluid bolus and 79% of them received appropriate analgesia for pain crises. Approximately 41.5% of the patients with fever were admitted and received ceftriaxone as single intravenous antimicrobial agent. However, none of the patients had bacteremia. Only 2.4% of the patients had either urinary tract infection or osteomyelitis based on imaging.

ED management is a key factor in the successful management of patients with SCD in a timely manner by providing fluids, analgesia, and antibiotics. Adopting evidence-based guidelines and avoiding unnecessary admissions are suggested in clinically well patients with fever in the era of completed vaccination, antibiotic prophylaxis, and good access to care for patients with a clear viral infection focus.

## 1. Introduction

Sickle cell disease (SCD) is one of the most common autosomal recessive inherited blood disorders caused by a mutation in the sixth amino acid of the β-globin gene. It affects approximately 1 in 2,500 births, 100,000 individuals in the USA, and 300,000 new cases globally each year (1). SCD was first recognized by James Herrick in 1910. However, the first case in Saudi Arabia was reported in the eastern province in 1963 by Lehmann et al. (2). It is estimated that approximately 61,000 patients with SCD live in Saudi Arabia (3). In patients with SCD, pathological hemoglobin (HbS) forms rigid polymers when deoxygenated, giving red blood cells a characteristic sickle shape. Increased blood viscosity and cell adhesion to the vascular endothelium could cause intermittent vaso-occlusions. Vaso-occlusive (VOC) attacks due to SCD manifest with frequent painful crises (4).

Many studies conducted in North America, Europe, and the Middle East have evaluated children with SCD who presented to the emergency department (ED) with pain, which is a hallmark of SCD and the primary reason for hospitalization or visits to EDs (5). Although there is considerable variability in the way SCD pain is managed, the standard treatment protocol for painful episodes has been analgesia, rest, and hydration. Therefore, it requires frequent systematic pain assessments (determining severity, location, characteristics, and associated symptoms) and systematic adjustment of comfort measures, particularly analgesics (non-steroidal anti-inflammatory drugs or opioids) (6).

Additionally, fever has been studied in children with SCD, as they are considered at risk of severe invasive bacterial infections by encapsulated pathogens, including *Streptococcus pneumoniae* and *Haemophilus influenzae* (7). This is a well-known complication of functional asplenia that usually develops in the first 5 years of life (7). Comprehensive care for disease control and preventing invasive infections recommended by the National Heart, Lung, and Blood Institute includes guidelines for management. These comprise prophylactic penicillin, vaccination against pneumococcus and other encapsulated pathogens, early initiation of hydroxyurea, folic acid supplementation, and education regarding the need for seeking immediate medical attention for fever and other serious complications (8–10).

Studies have evaluated the prevalence of SCD in different regions of Saudi Arabia, the characteristics of acute SCD complications, and the different phenotypes of disease manifestations (11–13). A multicenter prospective study included EDs from the United States and Canada that examined the adherence to 2014 National Heart, Lung, and Blood Institute guidelines in managing patients with SCD who presents VOC showed that fewer than half of the patients with VOC received parenteral opioids within the recommended time. Haven said that based on international literature examining the adherence to guidelines locally, in-depth EDs practice needs further exploration with the existing literature paucity(reference) (14).

Literature reviews for locally published articles need to be more comprehensive in the in-depth evaluation of current emergency practices in managing patients with SCD. The study aims to assess the current emergency practice in managing SCD patients in tertiary hospitals. Moreover, this study will also evaluate the prevalence and risk factors of invasive bacterial infections and VOC in patients who presented to ER with febrile illness at King Abdullah Specialized Children's Hospital, Riyadh, Saudi Arabia, over three years.

## 2. Materials and methods

This retrospective cross-sectional study included all children <14 years of age (cut age limit of treatment for care in the Department of Pediatrics) with SCD who presented to the emergency room with pain or fever at King Abdullah Specialized Children's Hospital, Riyadh, Saudi Arabia between January 2017 and December 2019.

The nature of the study was mainly descriptive to the current practice and prevalence estimation. Therefore, all episodes in pediatric patients diagnosed with SCD up to 14 years of age who presented to our ED during the study duration with fever or pain crises were reviewed. Patients who presented to the ED after bone marrow transplantation were excluded from the study.

### 2.1. Data collection

Sickle cell disease patients were identified using the patient electronic health information system (BestCare). All patients' diagnosis is coded in the system using the International Classification of Diseases, Tenth Revision (ICD-10) over the study period. Patients with SCD are coded in the system based on hemoglobin electrophoresis, the result of the newborn screen. All clinical patient information from Emergency visits was collected using a data collection sheet. Data on age, sex, season of presentation, vaccination status, penicillin prophylaxis, hydroxyurea treatment, patient disposition, vital signs at ED admission, and level of triage based on the Canadian Triage and Acuity Scale were collected (15). Data on route and timing of analgesia for pain crisis and antibiotics administration and timing for fever were also collected. We also collected data on the results of respiratory panel (multiplex polymerase chain reaction), blood, urine, or cerebrospinal fluid culture in cases of febrile episode. Data on presence of splenic sequestration crises, aplastic crises or hemolytic crises, and admission to hospital or intensive care unit were also analyzed. Sickle cell crises were defined as per the 2014 evidence-based guidelines (16). Splenic sequestration was defined as sudden enlargement of the spleen and a reduction in hemoglobin concentration by at least 2 g/dl below the baseline value. Acute aplastic crisis was defined as an acute decline of 2.0 g/dl or more in hemoglobin concentration below the patient's baseline value, with low reticulocyte count. Acute hemolytic crisis was defined as an acute decline of 2.0 g/dl or more in hemoglobin concentration below the patient's baseline value with high reticulocyte count and clinical evidence of hemolysis (17). Data were initially collected, coded, cleaned, entered into Microsoft Excel, and then analyzed using IBM Statistical Package for the Social Sciences (SPSS) version 21. The missing data was defined as non-serious into a random pattern and less than 5%.

We collected the study data for SCD patients who visited King Abdullah Specialist Children's Hospital (KASCH) Pediatric Emergency Department. The Pediatric Emergency Department is a 24-hour operating, seven-days-a-week consultant base practice that provides advanced medical care and a level I trauma center with a capacity of 50 beds. Annual pediatric visits have ranged from 80,000–100,000 in the last five years. The target served population is National Guard dependent and extends service to include Non-National Guard Citizens /non-Citizens who require specialty and emergency treatment. All patients who visit the Pediatric Emergency department will go through a standardized registration and triage to assign patients according to condition urgency. The hospital adopted the Canadian Triage and Acuity Scale-2012 (CTAS), a reliable tool to assign patient acuity based on a collection of subjective and objective information on all patients. Each patient assigned to acuity scores prioritizing patients with triage level 1 as resuscitation, triage level 2 as emergent, triage level 3 as urgent, triage level 4 as less urgent, and triage level 5 as non-urgent.

Descriptive analysis such as mean (standard deviation), frequency (%), tables, and graphs were used to describe the data. The chi-square test was used to examine the association between the outcome VOC and study variables such as gender, seasons, presence of fever/hypoxia, and the use of disease-modifying therapy. Logistic regression was used to determine the predictors of invasive bacterial infection and risk factors for acute pain crisis. *P*-values less than or equal to 0.05 were considered statistically significant.

## 3. Results

**Figure 1:**
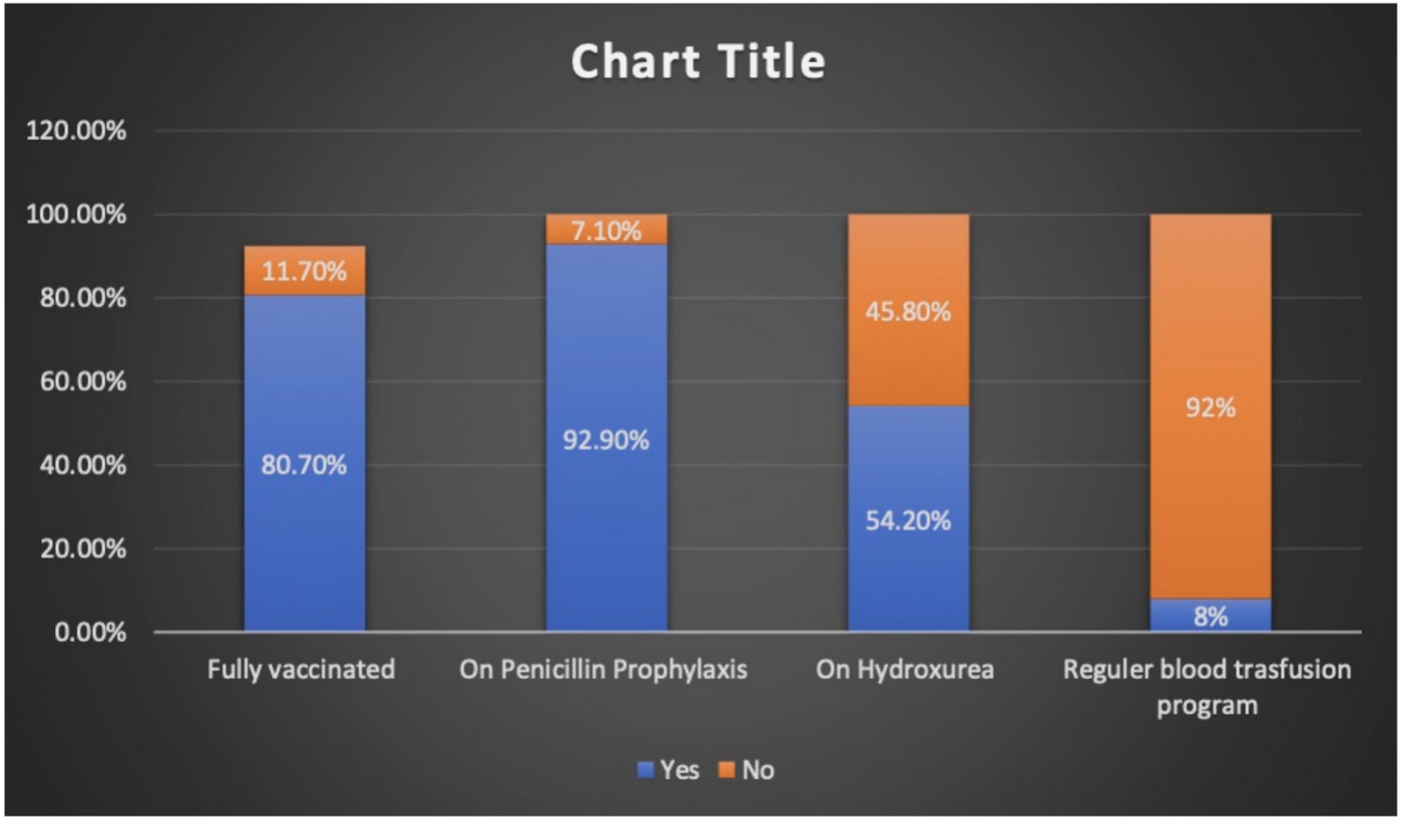
Percentages of patients with SCD on preventive and disease modifying therapy.

Charts of a total of 212 patients were reviewed in our study, and both sexes were equally represented. The median age at presentation during the study period was 7.6 years (IQR 4.2–10.8). The patients who presented with fever alone, pain alone, and fever and pain accounted for 37.7%, 47.2%, and 15%, respectively. The majority of hospital visits were in the fall and winter. The patients' blood groups were A^+^ in 41.5% and O^+^ in 46.2% of the patients. Based on their clinical condition upon presentation, the patients were triaged according to the Canadian Triage and Acuity Scale: 89% and 10% of the study cohorts were triaged as level III and level I and II, respectively (Table 1). Vaccination per recommendation was achieved in 80% of the patients, and 92.9% of them were on penicillin prophylaxis. The use of disease modifying therapy, such as hydroxyurea, was reported in 45.8% of the study participants (Figure 1).

**Table 1:** Demographics and clinical characteristics.

	*N*	Range or (%)
**Median Age**	7.6	(IQR 4.2,10.8)
1–5 years	92	(43.4)
6–10 years	71	(33.5)
11–15 years	49	(23.1)
Gender		
Male	104	(49.1)
Female	108	(50.9)
Seasons of presentation		
Winter (December 1–February 29)	69	(32.5)
Fall (September 1–November 30)	66	(31.1)
Spring (March 1–May 31)	37	(17.5)
Summer (June 1–Augest 31)	40	(18.9)
Vital signs at presentation		
Temperature	37.22	(36–40)
Heart Rate/min	122	
Based age on age/year.		
1–2 years	138	
3–5 years	135	
6–11 years	113	
12–15 years	107	
Respiratory rate/min. Mean Respiratory rate/min based on age /year.	28	
1–2 years	29	
3–5 years	30	
6–11 years	26	
12–15 years	26	
Oxygen saturation **%**	96%	
**Median HbS level**	70	Mode = 82 (reflects HBSS variant) or SBthal+
**Type and screen:**		
A	88	(41.5)
B	4	(1.8)
AB	9	(4.2)
O	111	(52.3)
Rh +/−	197/15	(93/7)
Presenting complaint		
Fever	80	(37.7)
Pain	100	(47.2)
Fever and pain	32	(15.1)
Sickle cell disease crisis and age distribution		
**VOC** ^a^	114	(53.7)
1–5 years	43	(37.7)
6–10 years	33	(28.9
11–15 years	38	(33)
s**ACS**^b^	13	(6.1)
1–5 years	5	(38.5)
6–10 years	4	(30.8)
11–15 years	3	(30.8)
**ACS and VOC**	9	(4.2)
1–5 years	1	(11)
6–10 years	5	(55)
11–15 years	3	(33.3)
**Splenic sequestration**	7	(3.3)
1–5 years	6	(85.7)
6–10 years	1	(14.3)
11–15 years	0	0
**Aplastic crises**	3	(1.4)
1–5 years	2	(66.7)
6–10 years	1	(33.3)
11–15 years	0	0
**Acute Hemolytic crises**	3	(1.4)
1–5 years	0	0
6–10 years	3	(100)
11–15 years	0	0
**Admission rate**	209	98
**Pediatric intensive care admission rate**	3	1.4
**Length of ED stay (hours)**	4	(1–9)
**Length of Hospital stay (days)**	5	(1–21)

^a^ Vaso- Occlusive crisis.

^b^ Acute Chest Syndrome.

### 3.1. Types of sickle cell disease crisis

**Figure 2:**
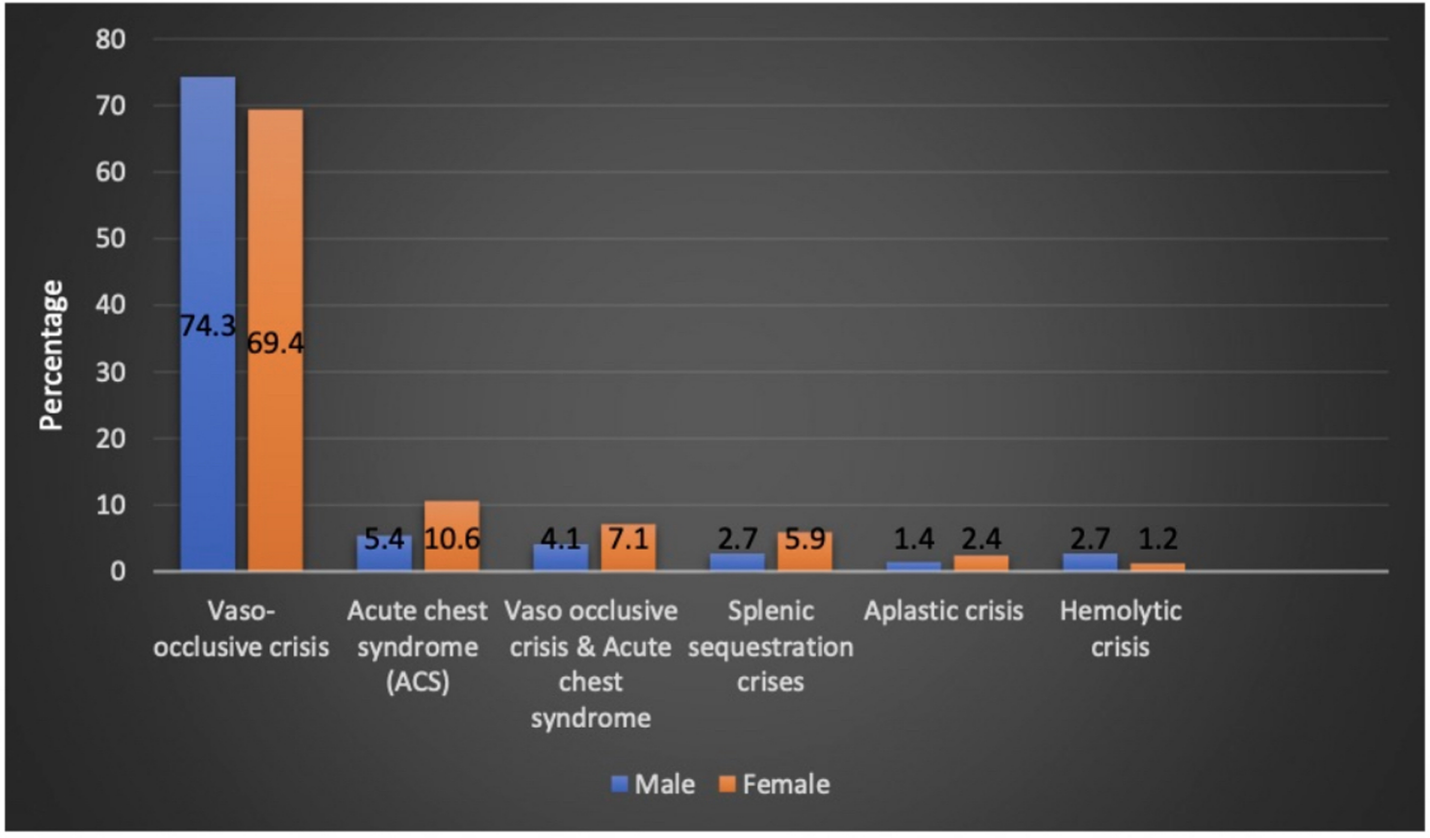
Sickle cell disease crisis and sex distribution.

The patients who had VOC crisis accounted for 53.8% of the patients during admission. Acute chest syndrome (ACS) was diagnosed in 6% of the study cohort. Acute sequestration crisis was observed in 3.3% of the patients. Aplastic and hemolytic crises accounted for 1.4% of the patients, each. VOC, ACS, and splenic sequestration crisis were seen more commonly in girls (Figure 2). SCD crisis distribution based on patient age is shown in (Table 1). In the 1–5 year-age group, 85.7% and 66.7% of the patients had splenic sequestration crises and aplastic crisis, respectively. However, significant hemolytic crisis was reported solely in the 6–10 year-age group.

### 3.2. Management in emergency department and admission to hospital

**Figure 3:**
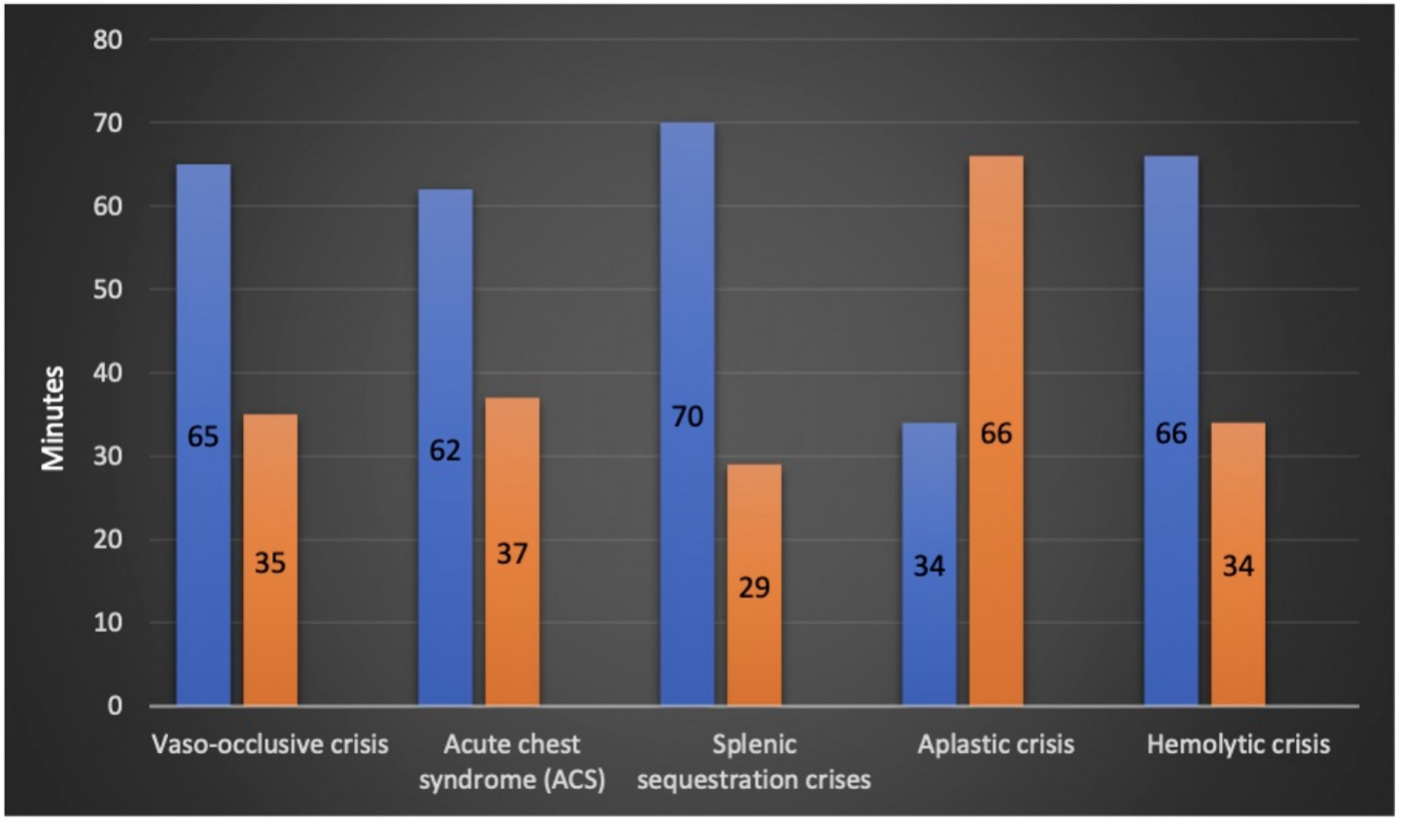
Time to be seen by emergency physician in minutes based on sickle cell disease crisis.

The mean time to be seen by a physician after arrival to emergency was 28 min, a maximum of 158 min was reported in one patient (0.5%). In the study cohort, 25%, 50%, and 75% were seen within 12, 22, and 37 min, respectively. Within 30 min, 66% of the patients were seen and assessed by ED physicians (Figure 3).

Regarding ED initial management, 50% and 36% of the patients received a bolus within one and two h post ED arrival. In the presence of pain crises, 50% and 79% of the patients received analgesia within one and two h post ED. In cases of fever, the first dose of antibiotics administration was given within 1 h in 20% of the patients and in 26% within the following hour post ED arrival. Of the study cohort, 98.6% were admitted to hospital to regular ward, and none of the patients required direct intensive care unit admission. However, during admission, 1.4% of the patients required transfer to intensive care unit. Direct patient discharge from the ED was in only 1.4% of the patients. The average length of ED stay was 4 h, and the average length of hospital stay was 5 days.

### 3.3. Pain crises management

Pain was the main presenting complaint in 64.6% of the patients. Of 64.6% of the participants, 13.8% had numerical pain score at triage above 5. Pain management was initiated in the ED within two h post arrival in 46.7% of the patients. Morphine sulfate was the most commonly prescribed pain medication given to 39.6% of the patients, followed by ibuprofen, which was given to 33% of the patients, and the remaining 20% received acetaminophen. Twenty % of the study cohort had received one dose of morphine in the ED, 16% required two doses, and 2.8% received three doses of morphine sulfate prior to admission. VOC crisis was the diagnosis and cause of pain.

### 3.4. Risk factors of VOC crisis in children with sickle cell disease

VOC crises were reported higher in girls (52.8%) compared with male patients (47.2%). Winter and fall seasons were reported to be higher compared with spring and summer, 32.5% and 31.1%, respectively, without statistical significance. The patients who exhibited hypoxia or oxygen saturation less than 95% and diagnosed with VOC crisis were in 10.5% of the patients without statistically significant risk. Fever was found to have a six-fold higher risk of being associated with VOC crisis (*P* = 0.001) (Table 2).

**Table 2:** Bivariate analysis of risk factors of Vaso–Occlusive crisis in children with sickle cell disease.

Study variables	*N* (%) Total 123	Odds ratio, 95% CI	*P*-value
Gender^a^		OR 0.424	0.09
Female	65 (52.8%)	95% CI	
Male	58 (47.2%)	(0.515–0.578)	
Seasons^b^		OR 0.555	0.115
Winter	40 (32.5%)	95% CI (0.241–1.279)	
Fall	38 (31.1%)		
Spring	22 (17.5%)		
Summer	23 (18.9%)		
Hypoxia^a^		OR 1.998	0.82
Oxygen Sat <95%	13 (10.8%)	95% CI (0.915–4.36)	
Oxygen Sat = or >95^%^	110 (89.4%)		
Fever^a^		OR 6.8	^``^0.001*
More than 38	18 (14.6%)	95% CI (3.56–13.1)	** **
Less than 38	105 (85.4%)		** **
Hydroxyurea^a^		OR 0.277	**0.001** *
Yes	72 (58.5%)	95% CI (0.154–0.497)	** **
No	51 (41.5%)		** **

Bold values means statistical significance.

^a^ Chi square.

^b^ Logistic regression for multivariate variable.

* Statistically significant *P*-value.

Hydroxyurea was found to be preventive for VOC crisis with statistical significance (*P =* 0.001) (OR, 0.277; 95% CI: 0.154–0.497).

### 3.4. Fever management

In the study cohort, 52.8% of the patients presented with documented fever to the ED. However, blood cultures were obtained from 174(82%) of the patients. Empiric antibiotics were administered in 52% of the patients (100% of all those with fever) using single agent 3rd generation cephalosporin (ceftriaxone) in 41.5% of the patients. Combined antibiotics were used in 10.3% of the patients.

Blood culture yielded no growth in any of the study participants. Urine culture was performed in 52.1% of the study participants, in which 2.3% were positive out of all urine culture. *Escherichia coli* accounted for 60% of urine bacterial growth and *Klebsiella pneumoniae* in 40% of the urine bacterial growth.

**Figure 4:**
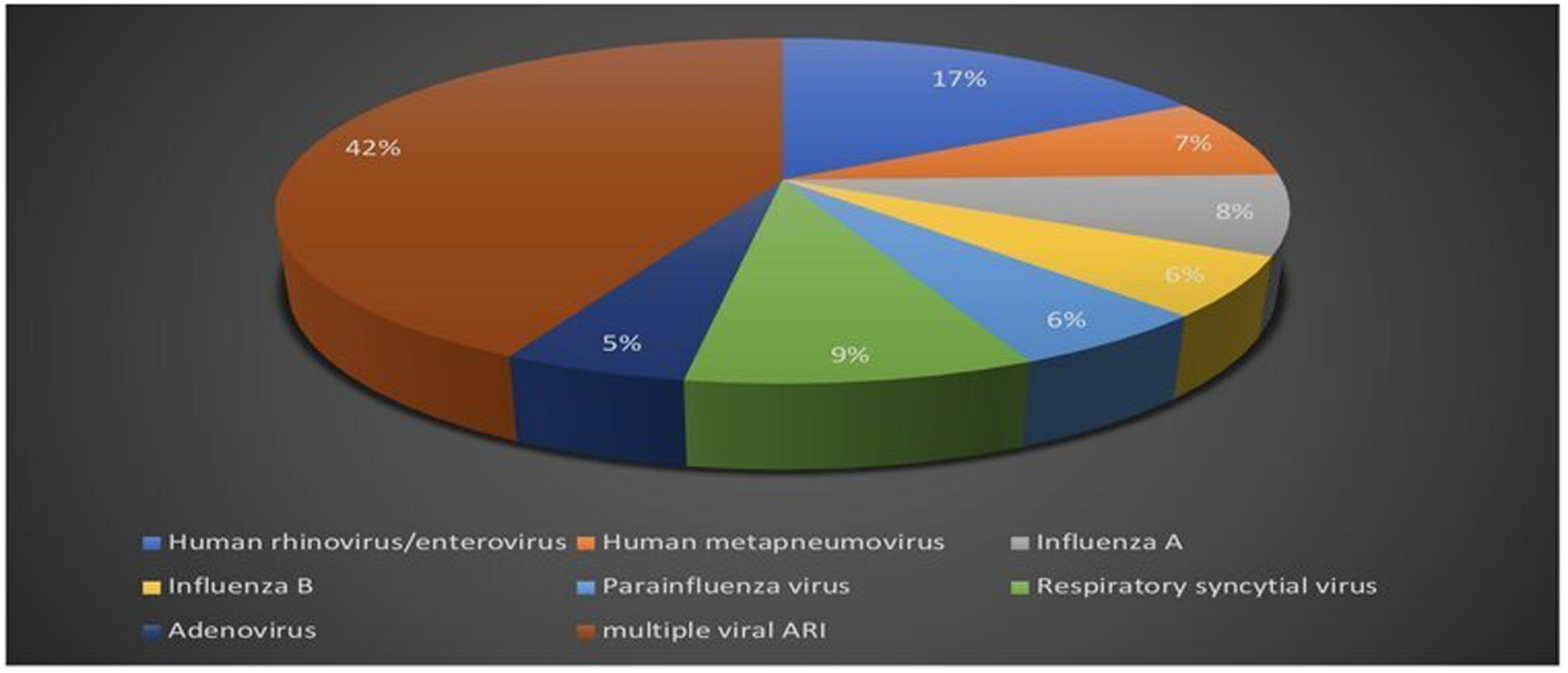
Respiratory viral multiplex polymerase chain reaction (PCR) test results.

Respiratory viral multiplex polymerase chain reaction test was obtained in 47.6% of the patients out of the total study population. Positive polymerase chain reaction testing was reported in 25% of the patients, single viral detection was reported in 57.6%, and multiple viral acute respiratory infections accounted for 42.4% (Figure 4).

A total of 124 patients underwent chest radiography during their hospital stay. A positive finding for new pulmonary infiltration was reported in 36% of the patients. Positive chest radiography was reported in 77% of the patients with ACS.

The prevalence of invasive bacterial infection was detected in 2.8% of the study participants: five patients (2.4%) had urinary tract bacterial infection and one (0.4%) was diagnosed with culture negative osteomyelitis. Due to the low prevalence of invasive infections, other risk factor variables were inconclusive.

## 4. Discussion

Our study described the current ED practices and identified related factors in the management of acute pain crises and febrile episodes in children SCD at our center over three years. This review showed the importance of timely management of SCD with fluids, antibiotics, and analgesia.

The distribution of patients and type of manifestation, such as pain, fever, ACS, splenic sequestration, and other crises were more representative of the African Haplotype SCD rather than of the Arab-Indian haplotype. This represents most likely patients from the southern region of Saudi Arabia rather than the eastern region. The age distribution of some complications was expected based on published data, such as splenic sequestration, which is more common in patients aged <5 years.

Most people with SCD experience VOC at some point in their lives (18). In our study pain crisis was the most common manifestation of SCD around 53.8%, particularly in girls in winter, similar to findings in previously published reports. Most patients received morphine sulfate followed by ibuprofen, as the literature supports the use of non-steroidal anti-inflammatory drugs as adjuncts in reducing pain and length of stay (19). VOC crisis was less frequent in the study cohort of patients using hydroxyurea. Previous studies have reported that hydroxyurea ameliorated the course of sickle cell anemia and was found to be effective in reducing the rate of VOC, as well as safe and well tolerated by the pediatric population. In addition, it has been reported that the use of hydroxyurea could reduce hospitalization and positively impact lives of children with SCD (20–22). In our study, we found that with the aggressive management which includes admission and antibiotics for patients with febrile SCD, the incidence of bacteremia was nil in the study cohort. Serious bacterial infections were reported in urine cultures: 2.3% of the patients had positive results of urine cultures. This is comparable to a study in patients with SCD that showed bacteremia in 1.7% of them (23). Another study showed a low prevalence of invasive infections, except when it was associated with an absolute neutrophil count >20 × 109/L with band cell (24, 25). Nevertheless, well-appearing SCD with fever can be managed in outpatient department after obtaining laboratory tests with the first dose of antibiotics (26), with outpatient department treatment reported to be cost-effective (27). Seasonal viral illness was found in this study as a triggering factor for VOC and ACS in up to 25% of patients in the study cohort. This finding is similar to those of previous studies, where respiratory syncytial virus infections were common in children with SCD and were associated with a higher risk for ACS and higher hospitalization rate (28, 29).

With a focus on ED management, more than half of the patients in the study cohort were seen by emergency physicians in less than one h and the majority was managed within a h window in the emergency room. In patients with VOC and pain, almost half received the first dose of analgesic, and almost 80% received pain management within 2 h. The American society of hematology 2020 guidelines for sickle cell anemia management of acute and chronic pain (30) recommend assessment and initiation of analgesic within one h, the study finding is keeping up with recommendation. However, multiple factors may play a role in passing the one h window such as ED crowding. Overall, the emergency management of the patients with SCD in this cohort in terms of fluid resuscitation, analgesic, and broad-spectrum antibiotics was found to be consistent with the guidelines and recommendations. However, the impact on hospitalization rate could not be assessed as almost all study participants were admitted to the hospital. Outcomes in the patients with SCD such as intensive care unit admission were extremely low in this cohort, which might limit the ability to evaluate the impact of emergency management (30, 31). As well as the impact of management, such as the use of fluid boluses, indicated in this review that most patients received fluid boules in the first two h. The implications for pain control and admission rate on the study cohort were not examined due to the nature of retrospective data collection, even though recent literature findings suggest fluid boluses in the first h are associated with poor pain control in patients with VOC (32).

This study has several limitations that may affect its generalizability. A retrospective chart review was used as a source of data, which might have limited information availability and confined the analysis to the data recorded on the patients' charts. Although this study was a single-center study with a small sample size, it provided an epidemiological review of SCD manifestations and emergency management that could be used as a baseline for future studies. Future studies and guideline adaptation are needed to avoid unnecessary admissions and assess the impact of emergency management and outcomes in patients with SCD, such as hospitalization and complication rates.

In conclusion, this study provided a single-center epidemiological review focusing on the emergency management service provided. The study findings may support future studies and guideline establishments. The study also evaluated the rate of invasive bacterial infection and VOC in patients with SCD seen in ED with risk prediction. Viral infection manifested by fever was found to be a risk factor for VOC. Whereas, using disease-modifying agents, such as hydroxyurea, reduces the risk of VOC in patients with SCD.

## Data Availability

The original contributions presented in the study are included in the article, further inquiries can be directed to the corresponding author.
